# Raman Study on Lipid Droplets in Hepatic Cells Co-Cultured with Fatty Acids

**DOI:** 10.3390/ijms22147378

**Published:** 2021-07-09

**Authors:** Pradjna N. Paramitha, Riki Zakaria, Anisa Maryani, Yukako Kusaka, Bibin B. Andriana, Kosuke Hashimoto, Hiromitsu Nakazawa, Satoru Kato, Hidetoshi Sato

**Affiliations:** 1 School of Biological and Environmental Sciences, Kwansei Gakuin University, 2-1 Gakuen, Sanda 669-1337, Hyogo, Japan; dgf88760@kwansei.ac.jp (P.N.P.); riki15001@mail.unpad.ac.id (R.Z.); anisamaryani1501@gmail.com (A.M.); yuts24a.lemonbalm@gmail.com (Y.K.); ftc87897@kwansei.ac.jp (B.B.A.); k-hashi@kwansei.ac.jp (K.H.); 2 Department of Biology, Universitas Padjadjaran, Jl. Raya Bandung Sumedang KM 21, Jatinangor, Sumedang 45363, Indonesia; 3 School of Science, Kwansei Gakuin University, 2-1 Gakuen, Sanda 669-1337, Hyogo, Japan; nakazawa@kwansei.ac.jp (H.N.); sk@kwansei.ac.jp (S.K.)

**Keywords:** Raman spectroscopy, biomedical, lipid, lipid droplet, electron microscope

## Abstract

The purpose of the present study was to investigate molecular compositions of lipid droplets changing in live hepatic cells stimulated with major fatty acids in the human body, i.e., palmitic, stearic, oleic, and linoleic acids. HepG2 cells were used as the model hepatic cells. Morphological changes of lipid droplets were observed by optical microscopy and transmission electron microscopy (TEM) during co-cultivation with fatty acids up to 5 days. The compositional changes in the fatty chains included in the lipid droplets were analyzed via Raman spectroscopy and chemometrics. The growth curves of the cells indicated that palmitic, stearic, and linoleic acids induced cell death in HepG2 cells, but oleic acid did not. Microscopic observations suggested that the rates of fat accumulation were high for oleic and linoleic acids, but low for palmitic and stearic acids. Raman analysis indicated that linoleic fatty chains taken into the cells are modified into oleic fatty chains. These results suggest that the signaling pathway of cell death is independent of fat stimulations. Moreover, these results suggest that hepatic cells have a high affinity for linoleic acid, but linoleic acid induces cell death in these cells. This may be one of the causes of inflammation in nonalcoholic fatty liver disease (NAFLD).

## 1. Introduction

Obesity is an important medical issue. A symptom of fat accumulation, especially in the liver in the absence of excessive alcohol consumption, is referred to as nonalcoholic fatty liver disease (NAFLD) [1]. When fibrosis progresses in the NAFLD liver, the liver progresses to nonalcoholic steatohepatitis (NASH), which greatly increases the possibility of liver cancer [1,2]. Adipocytes are able to hold a large amount of fat; while hepatic cells are unable to hold much fat. Medical studies strongly suggest that fat deposition in the liver is a major cause of the disease [1,3]. Fats in foods are converted into fatty acids and absorbed in the bowel. Fats are also synthesized in the body. Fatty acids are carried by lipoproteins, such as albumin, in the blood and passed to adipocytes in adipose tissues. In the fat metabolism of the body, when insulin signaling is reduced, adipocytes release fatty acids into the blood; subsequently, other cells, including hepatocytes, take up the fatty acids [3,4]. The previous studies found that NAFLD patients had an increased circulating lipid that is a symptom commonly found in people with obesity. Impaired visceral adipose tissue is a significant risk factor for NAFLD because it releases free fatty acids (FFAs) and adipocytokines that are directly delivered to the liver through the portal vein [5,6,7]. Fat accumulation in hepatocytes has been well studied using model cells [8,9,10,11]. Although there are many fatty acids with different chain lengths and numbers of double bonds in a chemical sense, the relatively small numbers of fats are important in edible fat studies, especially in animals. The major fat species accumulated in mammary animals, especially humans, possess fatty chains with 16–18 carbons and 0–2 double bonds [12,13]. The reactions of hepatic cells to palmitic and oleic acids have been extensively studied [8,9,10,11]. In previous studies, saturated fatty acids were reported to induce apoptosis in hepatic cells. Ricchi et al. [9] reported that HepG2 cells cultured with palmitic acids died by apoptosis, but those cultured with oleic acid survived. In contrast, Zhang et al. [14] reported that palmitic and linoleic acids induced ER stress and apoptosis in H4IIE cells, which are hepatic-model cells.

We studied the fat accumulation rate in adipose tissues of a live hamster body via Raman spectroscopy in our previous study [15]. A ball-lens top-hollow optical fiber Raman probe (BHRP) with a quartz ball lens of 800 µm diameter was used to analyze fat composition in subcutaneous adipose tissues in a totally noninvasive manner. The hamsters were fed diets with trilinolein (TL) and tricaprin (TC) for 6 weeks, and their body fat was analyzed via Raman spectroscopy. The result suggested that TL has an accumulation rate approximately 10 times higher compared with TC in the adipose tissue; in other words, adipocytes accumulate relatively more fats with linoleic chains. This research suggested that different fat species included in the diet, with various chain length and number of double bonds, would be accumulated at a different rate in the adipose tissue. On the other hand, different fat species overloads give distinct effects to hepatocytes. The chemical composition of the adipocytes may therefore determine the progression of NAFLD because the abundance of free fatty acid (FFA) that are released from the impaired visceral adipose tissue depends on its fat composition. Thus, it is important to study change in fat components in the hepatocytes stimulated with high FFA concentration. Raman spectroscopy has been applied for fat analysis in the case of hepatic cells. Kochan et al. [16] and Szafraniec et al. [17] applied Raman imaging to analyze fat accumulation in cells of the hepatic tissues of mouse models. They successfully demonstrated the accumulation of lipid droplets and fats in hepatic cells. This indicates that Raman spectroscopy is a strong tool for fat monitoring in the body to control human health. The accumulation rate of unsaturated fatty acids in hepatocytes is higher than that of saturated fatty acids [18]. Raman analysis showed that unsaturated lipids were ubiquitously detected in hepatocytes cultured with palmitic acid. In contrast, only a small amount of saturated lipids was identified in hepatocytes cultured with oleic acid [18,19]. Schie et al. [20] studied fat uptake and accumulation in HepG2 cells cultured with palmitic and oleic acids via Raman analysis and coherent anti-Stokes Raman scattering (CARS) imaging. They reported that the cells cultured with oleic acid and oleic/palmitic acids (50/50%) had a high content of these fatty acids in their lipid droplets; moreover, the cells cultured only with palmitic acid did not adhere to the substrate after 24 h of cultivation and induced apoptosis.

Foods rich in linoleic fatty chains, such as grapeseed oil and soybean oil, are often considered as healthy vegetable oils. As humans are unable to synthesize linoleic fatty chains, it is necessary to take up lipids with two double bonds from food [21]. This may explain why adipocytes have a high affinity for linoleic fatty acids. However, the reaction of hepatocytes is different from that of adipocytes. Although there are many fatty acids with different chain lengths and numbers of double bonds in a chemical sense, the relatively small numbers of fats are important in edible fat studies, especially in animals. The major fat species accumulated in mammary animals, especially humans, possess fatty chains with 16–18 carbons and 0–2 double bonds [20,21].

Consequently, it is necessary to study the metabolism of linoleic acid as well as other fatty acids in hepatic cells to understand the mechanisms of NAFLD and NASH. In the present study, we observed HepG2 cells, which are a model of hepatic cells, cultured with palmitic, stearic, oleic, and linoleic acids via Raman spectroscopy and transmission electron microscopy (TEM) to investigate the morphological and chemical changes in the hepatic cells stimulated by those fatty acids. We assumed that there were two major pathways for fat accumulation in HepG2 cells. One is the re-synthesizing and/or modifying pathway in which the cells take up fats once and reconstruct their favorite fatty acids. The other is a direct accumulation pathway in which the fatty acid chains are maintained during the pathway and carried into the lipid droplets.

## 2. Results and Discussion

### 2.1. Microscopic Observations

**Figure 1:**
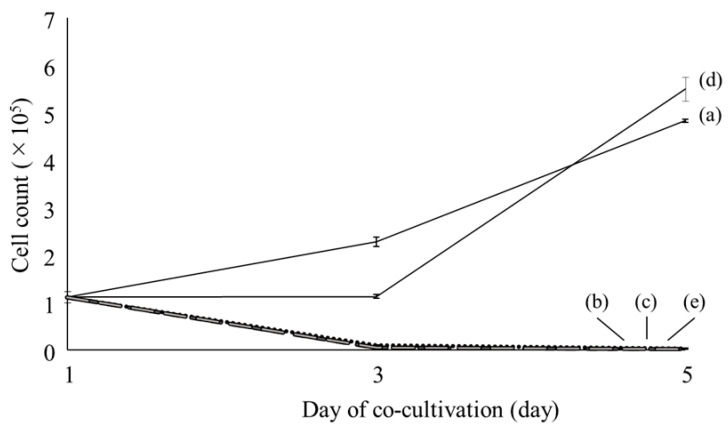
Growth curves of HepG2 cells cultured without fatty acid ((a); control) and with palmitic (b), stearic (c), oleic (d), and linoleic (e) acids.

HepG2 cells were cultured with bovine serum albumin (BSA) and four fatty acids, i.e., palmitic, stearic, oleic, and linoleic acids; the growth curves of these cells are shown in Figure 1. Albumin is a carrier of fatty acid chains, which makes the fatty acid soluble in the culture medium. Initially, 10^5^ cells were seeded in each dish. The number of live cells cultured with palmitic, stearic, and linoleic acids decreased down to 2000–3000 in their culturing dishes after 5 days. Ricchi et al. [9] suggested that saturated fatty acids induce apoptosis in hepatocytes. Curve (e) for the cells cultured with linoleic acid shows a reduction in the number of cells similar to that of the cells cultured with saturated fatty acids, although linoleic acid is an unsaturated fatty acid. Zhang et al. [14] reported that linoleic acid induces apoptosis in hepatoma cells, which seems to be the case in this study. In contrast, curve (d) for the cells cultured with oleic acid shows slow cell growth until day 3 under co-cultivation with fatty acid, followed by greater cell growth than that of control cells after 5 days. This suggests that oleic acid has a specific pathway for fat metabolism in HepG2 cells.

**Figure 2:**
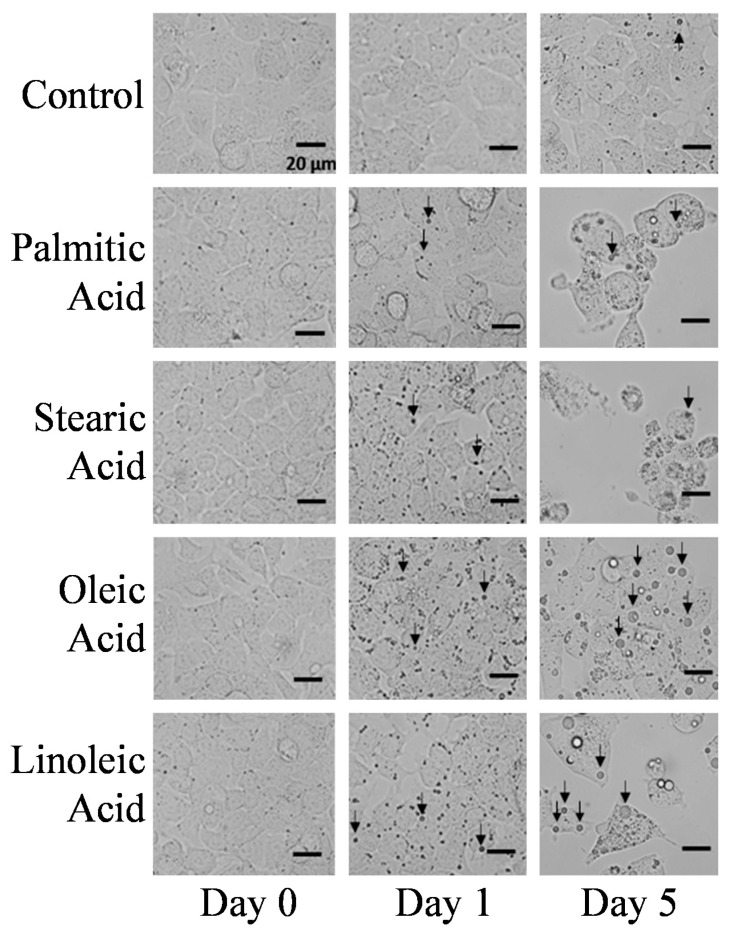
Optical microscopy images of HepG2 cells cultured without fatty acid (control) and with palmitic, stearic, oleic, and linoleic acids were observed on days 0, 1, and 5 of co-cultivation. Lipid droplets are indicated by arrows.

Figure 2 shows images of HepG2 cells observed on days 1 and 5 under cultivation with four fatty acids; the arrows indicate the lipid droplets in the cytoplasm. The lipid droplets appeared as small dark dots in the images. The images of control cells treated with a normal medium suggest that HepG2 cells generate few lipid droplets in the normal state. However, lipid droplets appeared at day 5, suggesting that lipid accumulation is an inherent function of HepG2 cells. The cells were seeded only 1 day before the beginning of fatty acid treatment; therefore, they seemed to possess less fat at day 1 because they needed energy for their normal metabolism. Cell passaging is an energy-consuming process because the cells must reconstruct their foot proteins to attach to the substrate. Furthermore, the cells started to propagate immediately after settlement. The images of cells cultured with stearic, oleic, and linoleic acid showed that the lipid droplets increased remarkably. The lipid droplets in the cells cultured with palmitic acid slightly increased, about twice more, on day 1. The lipid droplets grew in size at day 5 in the cells cultured with the four fatty acids. This suggests that the cells maintain a high rate of lipid uptake or excess generation even during the dying process. Adipose tissues and the liver are the main organs that are involved in the regulation of fat storage and metabolism to maintain the energy homeostasis. Adipocytes and hepatocytes highly express fatty acid transporter protein (FATP) [6]. The observed continuous fat accumulation in the hepatocytes may be a consequence of the high expression of FATP, caveolins, and fatty acid translocase (FAT/CD36) in the hepatocytes [22]. When the adipose tissue function is impaired, i.e., when inflammation and insulin resistance occur, the adipose tissue is unable to rescue the circulating FFAs. This may cause ectopic fat deposition which leads to organ damages [4], especially in the liver which has a high fat uptake capacity.

The average number and size of optically visible lipid droplets in the cells are listed in Table 1. The cells treated with palmitic and stearic acids changed their shape at day 5 because these fatty acids induced apoptosis, as indicated by the growth curves. In contrast, the cells treated with oleic acid showed no changes in their appearance, except for the increment in the number and size of the droplets. The cells treated with linoleic acid showed a somewhat different morphological appearance that did not resemble with that of the cells treated with palmitic acid, stearic acid, or oleic acid. As indicated by the growth curves, the cell growth was drastically suppressed by linoleic acid. The cells treated with palmitic and stearic acids showed bulges and bloating, which are typical features of apoptotic cells, but the cells treated with linoleic acid did not show these characteristic features by visual observation. Zhang et al. [14] reported that linoleic acid induces apoptosis in hepatoma cells, and the mechanism of apoptosis induced by linoleic acid is different from that induced by palmitic acid. The different appearances may be due to the apoptotic mechanism. As shown in Table 1, the particles of lipid droplets increased and grew in size, even in the apoptotic cells.

Average numbers of lipid droplets for the control cells were 0.20 and 0.27 for days 1 and 5, which indicated that one lipid droplet was found for every 4–5 cells. The cells treated with fatty acids possessed more and larger lipid droplets than those possessed by the control cells. The number and size of lipid droplets in cells treated with unsaturated fatty acids were higher than those of lipid droplets in the cells treated with saturated fatty acids. It should be noted that at day 5 of fatty acid treatment, the average number of lipid droplets for the cell treated with linoleic acid was lower than that treated with oleic acid, but the average size of the lipid droplets in the cell done with linoleic acid was bigger than the other. Smaller lipid droplets were significantly decreased after 5 days of treatment with linoleic acid. In contrast, small lipid droplets were still ubiquitously present in oleic acid treated cells. The lipid uptake system works more efficiently with unsaturated fats. These results strongly suggest that the pathway of lipid uptake seems to be completely independent from that of apoptosis. The values in Table 1 merely represent lipid droplets larger than approximately 1.75 µm because it was difficult to discern small lipid droplets from organelles. Furthermore, it is the smallest size suitable for the Raman analysis because the spatial resolution of the present Raman system is about 0.8 × 0.8 × 1.1 µm.

**Figure 3:**
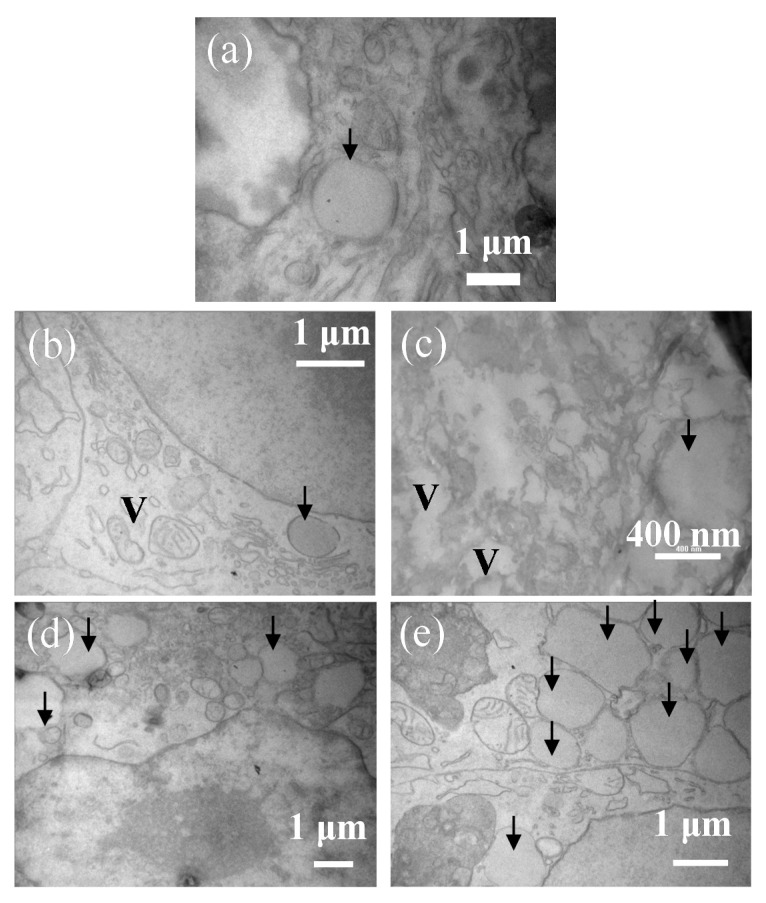
TEM images of HepG2 cells cultured without fatty acid ((**a**); control) and with palmitic (**b**), stearic (**c**), oleic (**d**), and linoleic (**e**) acids observed on day 5. Lipid droplets and vacuolations are shown with arrows and “V”.

TEM was employed to observe the distribution of small lipid vesicles that were not observable with the optical microscope and vacuolation which is usually observed in apoptotic cells. Figure 3 depicts the TEM images of HepG2 cells treated with the fatty acids for 5 days. The control cells possessed a much smaller number of lipid vesicles compared with those treated with the fatty acids. Moreover, the cells treated with unsaturated fatty acids possessed much more lipid vesicles compared with those treated with saturated fatty acids. This suggests that the cells cultured with oleic and linoleic acids contain a high density of fats in their cytoplasm. Extensive vacuolation was observed in cells treated with saturated fatty acids, and slight vacuolation was observed in cells treated with linoleic acid. These morphological changes suggest that these cells were unhealthy because of the induction of apoptosis by saturated fatty acids and linoleic acid. Fragmentation and/or blobbing of nuclear is observed also in the dying cells treated with linoleic acid, which is a typical reaction in the apoptotic cells [23] (Supplementary Figure S1). This strongly suggests that the linoleic acid stimulation induces apoptosis to HepG2 cell. In contrast, the cells treated with oleic acid did not exhibit vacuolation, and many vesicles filled the cytosol. TEM observation suggested that the lipid droplets were generated as small vesicles and grew by absorbing the small vesicles. In the TEM image of the cells treated with linoleic acid, there were a few vacuoles and many small vesicles filling the cytosol. In the optical microscopy image of the cells treated with linoleic acid, the appearance of the dead cell resembled necrotic debris, of which the cellular membrane had been destroyed and the organelle and lipid droplets leaked out. This suggests that the apoptotic cells with linoleic acid show a somewhat different appearance because the inner pressure exerted by the lipid droplets in these cells destroys the cellular membrane damaged by the apoptotic process.

### 2.2. Raman Spectroscopy Analysis

**Figure 4:**
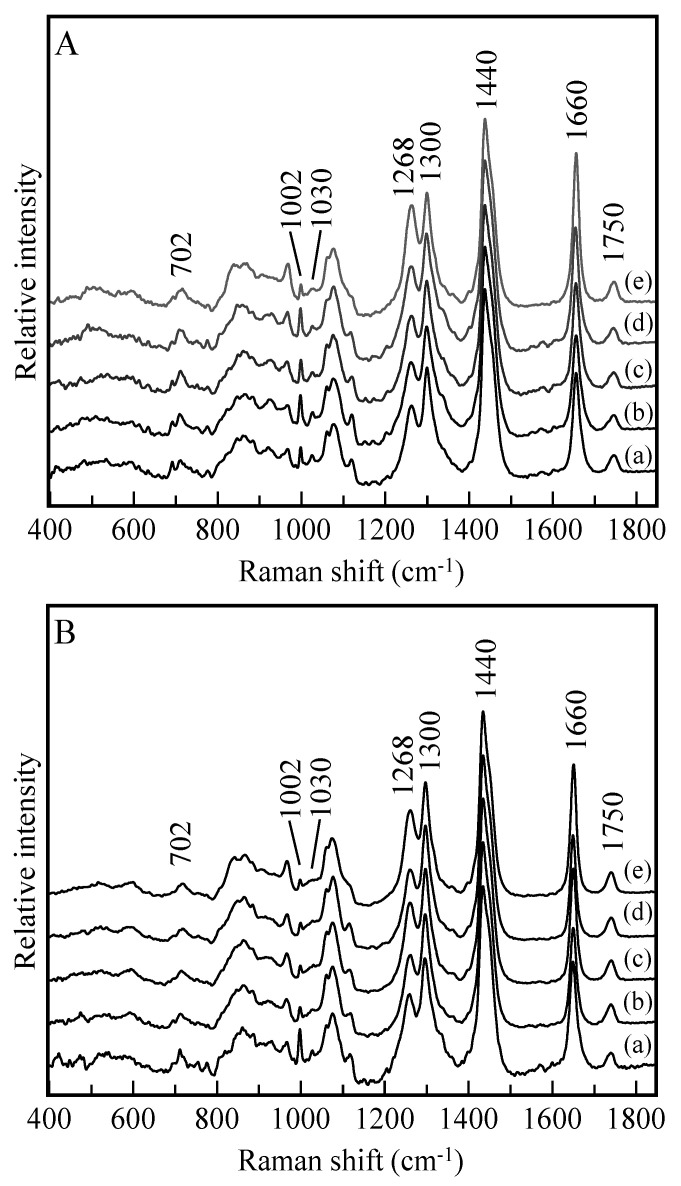
Averaged Raman spectra of lipid droplets measured on days 1 (**A**) and 5 (**B**). The spectra were obtained from the cells cultured without fatty acid ((a); control) and those cultured with palmitic (b), stearic (c), oleic (d), and linoleic (e) acids.

A Raman microscope was used to analyze the ingredients of lipid droplets in live HepG2 cells. As observed in a previous study on subcutaneous fat accumulation, there may be differences in the accumulation rates among the palmitic fatty acid-, stearic fatty acid-, oleic fatty acid-, and linoleic fatty acid-treated cells. We focused the excitation laser on lipid droplets that were visually observed but of various sizes to measure their Raman spectra. The measurements were conducted for 30 droplets in approximately 10 cells per dish. The spectral features (Supplementary Figures S2 and S3) obtained for each dish were similar to each other, suggesting that the molecular composition of the lipid droplets was homogeneous over the cells in each dish regardless of the size of the droplet. The averaged spectra of the droplets are compared in Figure 4. A band at 1750 cm^−1^, commonly observed in all the spectra, is assigned to the C=O stretching mode of the ester group of triacylglycerols. The band at 1750 cm^−1^ observed in all the spectra reveals that the spectra are highly contributed by the fat in the lipid droplets, which indicates that the cells take up the fatty acids and modify them into triacylglycerols for storage. In contrast, sharp bands at 1003 and 1030 cm^−1^ originate from the ring breathing modes of phenylalanine included in the protein species. The intensity of these bands is relatively low, especially in the cells treated with fatty acids for 5 days. As the droplet in the cell at day 5 is larger than that of the cell at day 1, the corresponding Raman spectra reflect the ingredients at the center of the droplet. This suggests that the lipid droplets have lipoproteins on their surfaces. The bands at 1660 and 1268 cm^−1^ are assigned to the vibrational modes of the C=C and H-C=C groups. Their intensities indicate the concentrations of the fatty chains with double bonds. The spectra of the cells treated with linoleic acid show strong bands. As linoleic acid has two double bonds in its chain, it can be inferred that the cells follow a pathway of triacylglycerol synthesis, which uses the fatty acid chains directly as they are harvested without modification. However, the reduction of the band intensities at 1660 and 1268 cm^−1^ is not observed in the spectra of the cells treated with saturated fatty acids. According to the visual comparison, the spectra of the cells treated with palmitic, stearic, and oleic acids are similar. As it is difficult to make a detailed analysis by comparing the spectral features because of the overlapping bands, a chemometrics analytical technique is applied to the spectra.

**Figure 5:**
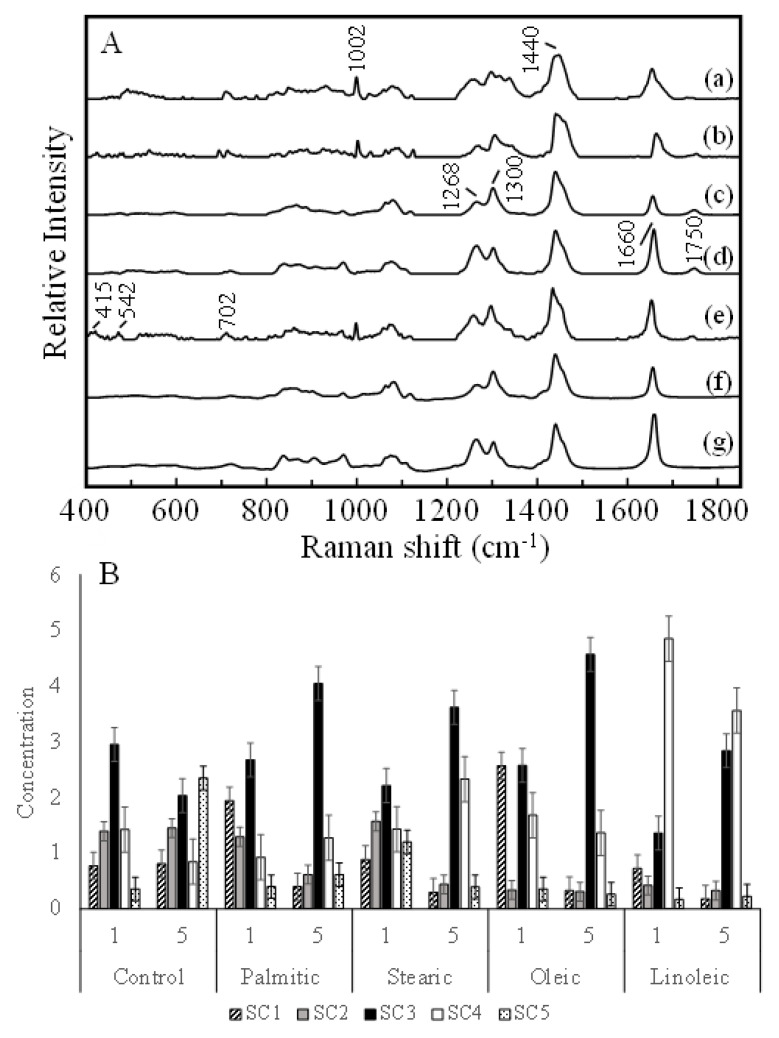
Results of MCR-ALS analysis. The five major components (a–e) obtained by MCR analysis (**A**) were compared with the spectra of pure oleic (f) and linoleic (g) acids. The ALS scores of the five components (**B**) are exhibited for the spectra of the cells cultured without fatty acids and those cultured with palmitic, stearic, oleic, and linoleic acids.

The spectra were analyzed via MCR-ALS analysis to investigate the molecular compositional changes in more detail. Figure 5A compares the spectral components (SCs) from 1 to 5 obtained by MCR analysis and the pure spectra of oleic and linoleic acids. SCs 1(a), 2(b), and 5(e) show bands at 1003 and 1030 cm^−1^ due to proteins, which indicates that these SCs include a large contribution from the protein species. SCs 2 and 5 exhibit a weak band at 1735 cm^−1^ due to the C=O stretching mode, suggesting that they also include contributions from lipids. Quantitative analysis based on SC1, 2, and 5 is not suitable because these components have mixed contributions from proteins and other cellular components. In contrast, the SCs 3(c) and 4(d) closely resemble the spectra of pure oleic (f) and linoleic (g) acids, respectively. The spectra of pure fatty acids lack a band near 1735 cm^−1^ and show a slightly stronger band near 1660 cm^−1^. Because they usually adopt a dimer structure in which the oxygen of the -C=O group is bound to the hydrogen of HO- by a hydrogen bond, the band due to the C=O stretching mode shifts far down and overlaps with a band due to the C=C stretching mode near 1660 cm^−1^ [24]. This strongly suggests that SCs 3 and 4 represent the oleic and linoleic fatty chains in the triacylglycerol species. The SC scores are shown in Figure 4B. In the control cells, the score plots of 1 and 5 days show variations, but the ratio between the scores of SC 3 and 4 (SC4/SC3) is maintained at approximately 0.5. This suggests that the rate of fat accumulation for oleic chains is approximately twice as high as that for linoleic chains. The ratio SC4/SC3 varies slightly in the cells cultured with palmitic, stearic, and oleic acids, which suggests that the cells synthesize oleic based on these fatty acids. Linoleic acid is an essential lipid that HepG2 cell is not synthesize by itself. The component of linoleic acid found in the control cell and cells treated with palmitic, stearic and oleic acids came from the culturing medium, probably from fetal bovine serum (FBS).

In contrast, the ratios SC4/SC3 are 3.0 and 1.2 for the cells cultured with linoleic acid for days 1 and 5, which are much larger than those of other cells. This indicates that the cells take up the linoleic chains and directly subsume into the triacylglycerol synthesis process. In our previous study, the fat accumulation rate was investigated in the adipose tissues of hamsters treated with TL and TC diets; the results suggested that the accumulation rate of TL was much higher than that of TC [15]. Because TL consists of linoleic fatty chains, the results of our previous study agree with the results of the present study. However, as discussed before, the linoleic fatty chain is not safe for hepatic cells, which may explain why the SC4/SC3 ratio decreased on day 5. This indicates that the cells modify one of the double bonds in the linoleic chains and generate oleic chains that are relatively safe for storage in hepatic cells. As discussed earlier, the metabolic system of the whole body and adipose tissue possess a high affinity for linoleic acid and a high linoleic acid accumulation rate. This suggests that linoleic acid does not induce apoptosis in adipocytes. In contrast, linoleic acid induces cell death in HepG2 cells, although the accumulation rate of linoleic acid is higher than that of palmitic, stearic, and oleic acids. The comparison suggests that adipocytes and hepatic cells have similar pathways for fat accumulation, but the pathway of cell death, which may be apoptosis, are completely different.

The results demonstrated that a high concentration of saturated fatty acids; palmitic and stearic acid, caused a lower degree of fat accumulation as lipid droplets in HepG2 cells. However, saturated FFAs can induce the activation of the intrinsic apoptosis pathway in hepatocytes [6]. On the other hand, HepG2 cells have a higher capacity to accumulate linoleic acid as lipid droplets compared to saturated fatty acids. However, this will also lead to hepatocytes’ cell death with a hypothetically distinct mechanism to those induced by saturated FFAs, as shown by the different appearance of the apoptotic cells. Impaired adipocytes release fat and hepatocytes take it up. The FFAs species released by the adipocytes may determine the severity of fat accumulation in the liver, and hence the progression of NAFLD. The results of this study may suggest one of the causes of NAFLD and NASH. In our previous study on in situ analysis of fat accumulation in the subcutaneous adipose tissue using Raman probe, we succeeded in quantitative analysis of fat components with different chain length and saturation [15]. Raman spectroscopy is capable to analyze fat ingredient in foods too [25,26,27]. The present technique, Raman spectroscopy with multivariate analysis, is commonly applied for foods, in vitro cell, and in situ tissue analyses. Hence, it will be a feasible tool for monitoring fat metabolism and health control.

## 3. Materials and Methods

### 3.1. Cell Culture

The HepG2 cell line, derived from human hepatoblastoma, was obtained from RIKEN BRC Cell Bank (RIKEN Bioresource Research Center, Tsukuba, Japan). HepG2 cells were cultured in low-glucose Dulbecco’s modified Eagle medium (DMEM; Thermo Fisher Scientific, Tokyo, Japan) supplemented with 10% fetal bovine serum (Biosera, Fuji-film Wako Chemicals, Osaka, Japan) and 1% penicillin/streptomycin (Thermo Fisher Scientific, Japan). The cells were cultured in an incubator with a 5% CO_2_ air flow at 37 °C. During cell culture, the medium was changed every three days. When the cells were treated with fatty acids, the medium was also changed to the new one with the same fatty acid contents. Four types of fatty acids, palmitic acid (16:0), stearic acid (18:0), oleic acid (18:1), and linoleic acid (18:2), were employed to induce steatosis in the cells. All pure fatty acids were purchased from Sigma-Aldrich Chemicals, Japan. Bovine serum albumin (BSA; Sigma-Aldrich Chemicals, Tokyo, Japan) was added to the medium for solubilization of the fatty acid in the medium prior to the addition of the fatty acid at a concentration ratio of 2:1 for the fatty acid to BSA [28]. The medium containing the fatty acid was shaken in a water bath at 37 °C overnight, sonicated with Handy Sonic (Tomy Seiko Co., Ltd., Tokyo, Japan), and filtered with a syringe filter unit (Millex-GV, 0.22 μm PVDF membrane, Merck-Germany, Tokyo, Japan). For live-cell Raman measurements, 1 × 10^5^ cells were seeded in a quartz-bottomed dish (Fine Plus International Ltd., Kyoto, Japan). After 24 h, the medium was replaced with fresh medium containing 100 μM fatty acid. The experiment was repeated 3 times. Deviations in the cell counting study are shown with error bars in Figure 1.

To study the detailed reactions of the cells, the concentrations of the fatty acids were regulated to take a several days for inducing cell death. In the preliminary experiments, the concentration of the saturated fatty acid varied from 50 to 500 µM. In the present condition, the HepG2 cell shows no or very few cell death at day 1 but shows remarkable reduction of its population at day 5.

Raman measurements were performed for each treatment group after one and five days of fatty acid treatment. The cells for cell counting were cultured under the same condition separately. The cell counting was carried out using hemocytometer and trypan blue staining (Trypan Blue stain 0.4%, Thermo Fisher Scientific, Japan).

### 3.2. Confocal Raman Micro-Spectroscopy

Raman measurements of the live cells were performed using an in-house-built confocal Raman system equipped with an inverted microscope (IX73, Olympus, Tokyo, Japan), a polychromator (grating: 600 L/mm, f = 320 mm, 750 nm blazed, Photon Design Co., Tokyo, Japan), a cooled CCD detector (DU401-BR-DD, Andor Technology, Oxford, UK), a diode laser (785 nm, Toptica, Munich, Germany), and a CO_2_ incubator for inverted microscope. The incubator was driven at 37 °C and 5% CO_2_ during the measurements. A water-immersed objective lens (×60, NA = 1.2, Olympus, Tokyo, Japan) was used for laser focusing and collection of Raman-scattered light. The approximated laser spot size was 0.8 μm in the lateral direction and 1.1 μm in the depth direction. The exposure time was 10 s × 3 times with a laser power of 60 mW at the sample point. Live cell Raman measurements using an excitation laser of 785 nm has been widely applied in live cell studies without damage to the irradiated cells [29,30]. The present condition was enough low to keep viability of the cell. Raman measurements were performed on each lipid droplet in the HepG2 cells. Thirty spectra (*n* = 3) of lipid droplets with various sizes were collected randomly from the monolayer cells of each treatment group.

### 3.3. Data Analysis

The measured spectra were processed before analysis using multivariate analysis. Background noise was subtracted to remove signals originating from the glass and culture medium. The spectra were subjected to spectral smoothing and baseline correction using the Savitzky–Golay method (nine points) and polynomial line fitting, respectively; moreover, the spectra were normalized with a band at 1440 cm^−1^ assignable to a CH vibrational mode. Multivariate curve resolution-alternating least squares (MCR-ALS) analysis was performed using chemometrics software (Unscrambler; CAMO, Bedford, MA, USA). The experiment was repeated three times independently. The spectra obtained in the three experiments were analyzed together. MCR-ALS analysis is one of the multivariate analysis techniques to predict pure component spectra in an unknown mixture and their relative abundance [31]. A test set of spectra of varied lipid droplets (matrix X) were decomposed into two matrices: concentration matrix (C) and pure CS matrix (S^T^). MCR analysis decompose a set of spectra into a several common spectral components (SCs). ALS analysis obtains the possible concentration of each SC in an unknown spectrum. The spectra were analyzed under non-negative constraints for the bands and concentrations. Furthermore, the spectra of pure palmitic acid (in liquid phase), stearic acid (in liquid phase), oleic acid, linoleic acid, and cholesterol were used as the initial guesses of the pure component spectra.

X → CS^T^

### 3.4. Transmission Electron Microscopy (TEM)

HepG2 cells (1 × 10^5^ cells) were seeded onto a 0.4 μm polycarbonate membrane (Transwell^®^ permeable support, 6.5 mm insert, 24-well plate, Corning Incorporated, New York, USA). After 24 h, the cells were co-cultured with 100 μM fatty acids for 5 days. Subsequently, the cells were fixed with 1% paraformaldehyde (FUJIFILM Wako Chemicals, Osaka, Japan) by immersing the membrane for 1 h. The membranes were washed three times with phosphate-buffered saline (PBS) and then immersed in a mixture (1:3) of 4% osmium tetroxide solution (Nisshin EM Co., Ltd., Tokyo, Japan) and 0.02% potassium hexacyanoferrate(II) trihydrate (Sigma-Aldrich Chemicals, Tokyo, Japan) for 2 h at 4 °C. Samples were dehydrated by serial washing with ethanol (50%, 70%, 80%, 90%, 95%, and 100%) and n-butyl glycidyl ether (QY-1, Nisshin EM Co., Ltd., Tokyo, Japan) three times. The samples were embedded in resin composed of araldite, hardener, and DMP-30 and subsequently dried in an oven at 60°C. The samples were cut into ultrathin sections (approximately 70–90 nm in thickness) using a Leica Ultracut UCT ultra-microtome, placed on Cu/Rh grids (Nisshin EM Co., Ltd., Tokyo, Japan), and contrasted using a 4% solution of neodymium (III) acetate (Sigma-Aldrich Chemicals, Tokyo, Japan) for TEM observation. Ultrastructural observation of the HepG2 cells was performed using an electron microscope (JEM-1400, JEOL, Tokyo, Japan) at an accelerating electron voltage of 100 kV. The TEM images were recorded using a Gatan camera controlled with the DigitalMicrograph™ software.

## 4. Conclusions

HepG2 cells are able to take up fat components directly from the cultivation media and use the fat directly or indirectly to synthesize triacylglycerol that accumulates in the lipid droplets. The pathways of fat uptake and lipid droplet generation are not regulated by the lipid concentration in the cells and seem to be free from apoptotic signaling pathways. HepG2 cells possess a special pathway for linoleic fatty-chain modification of oleic acid during fat metabolism. Raman spectroscopy is commonly applicable for analyzing lipids in biological tissues and in foods as well as in live cells. The multivariate analysis helps to extract semi-quantitative information of fat components from the Raman spectra in a totally noninvasive manner. The present result strongly suggests that the Raman spectroscopy with multivariate analysis is feasible for use in health control, especially by monitoring the fat metabolism in foods, adipose tissue, and liver which deeply relates to NAFLD progression.

## Figures and Tables

**Table 1:** Average diameter and number of lipid droplets found in the cells co-cultured with fatty acids.

	Treatment Time (day)	Average Number of Lipid Droplets/Cell	Average Size of Lipid Droplets
Control	1	0.20	1.88 ± 0.30
	5	0.27	2.10 ± 0.43
Palmitic acid	1	0.55	2.36 ± 0.51
	5	0.87	2.62 ± 1.43
Stearic acid	1	0.72	2.09 ± 0.72
	5	1.69	3.63 ± 2.20
Oleic acid	1	0.78	2.14 ± 0.48
	5	5.45	3.01 ± 1.52
Linoleic acid	1	1.09	2.51 ± 1.10
	5	3.63	4.47 ± 2.41

## Data Availability

Data are contained within the article and supplementary materials.

## Supplementary Materials

The following are available online at https://www.mdpi.com/article/10.3390/ijms22147378/s1.
