# Supplementary material for: Raman Study on Lipid Droplets in Hepatic Cells Co-Cultured with Fatty Acids
Source: Int J Mol Sci. 2021 Jul 9;22(14):7378. doi: 10.3390/ijms22147378 (PMC8307330; doi:10.3390/ijms22147378)
Supplement: Supplementary file 1 [file ijms-22-07378-s001.zip › ijms-1273531-supplementary.pdf]

Supplementary figures

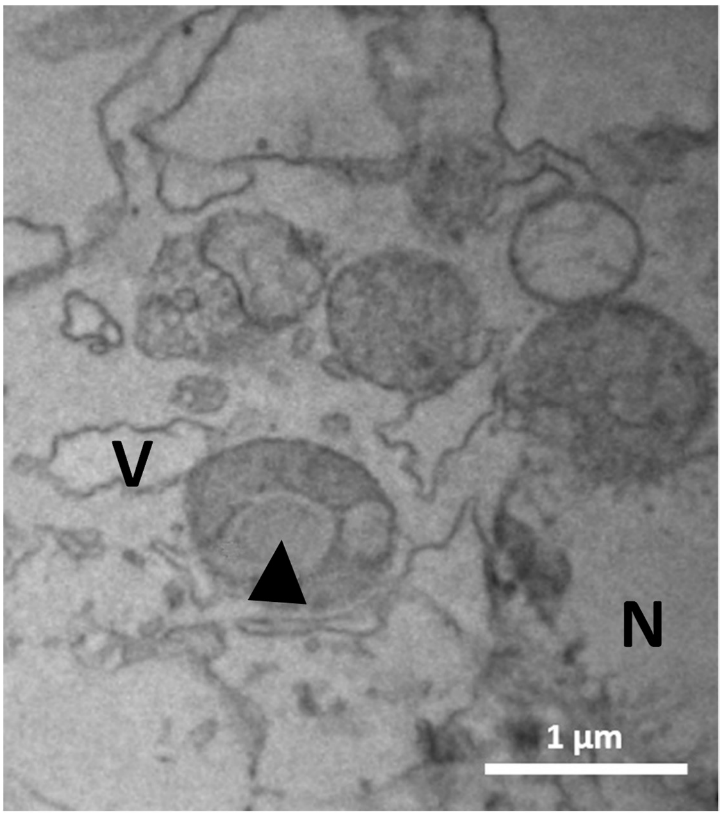

Supplementary Figure S1. TEM images of HepG2 cells cultured with linoleic acid, showing signs of apoptosis on day 5. Condensed and shrunk nucleus (arrowhead) were observed inside apoptotic bodies. N (nucleus), V (vacuole).

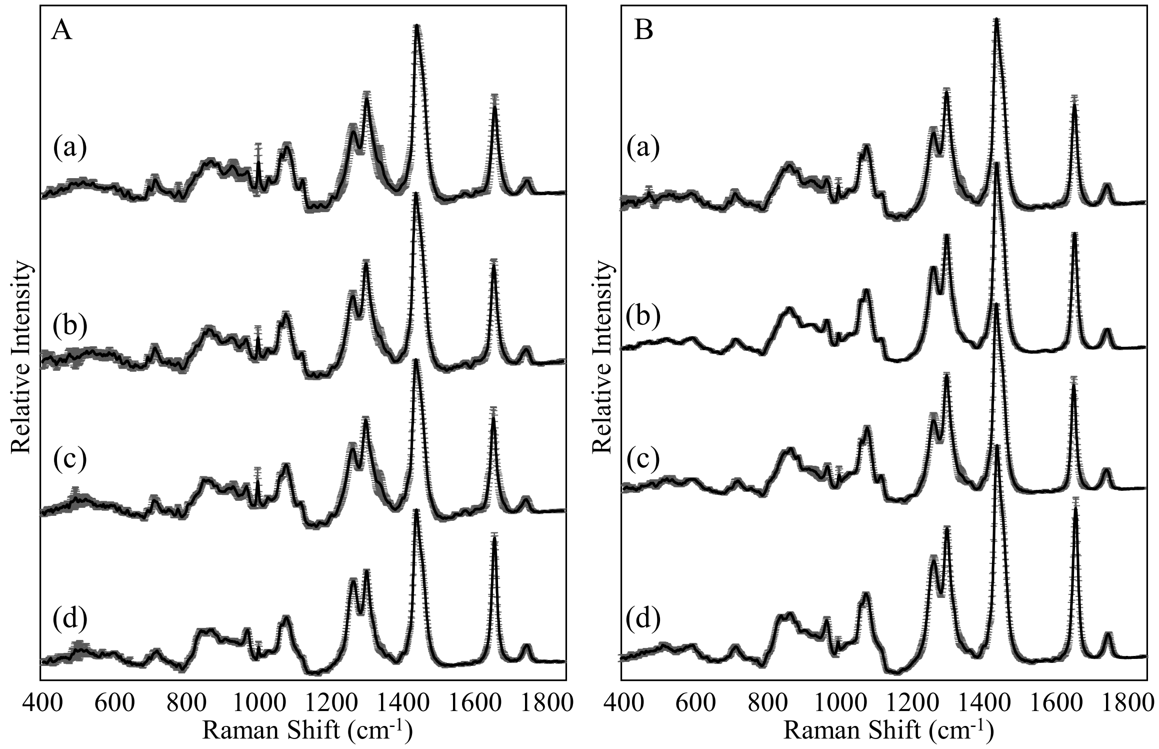

Supplementary Figure S2. Raman spectra of lipid droplets with error bar representing their standard deviations. (n=30×3 times) The spectra on day 1 (A) and day 5 (B) of the cells treated with palmitic (a), stearic (b), oleic (c) and linoleic (d) acids.

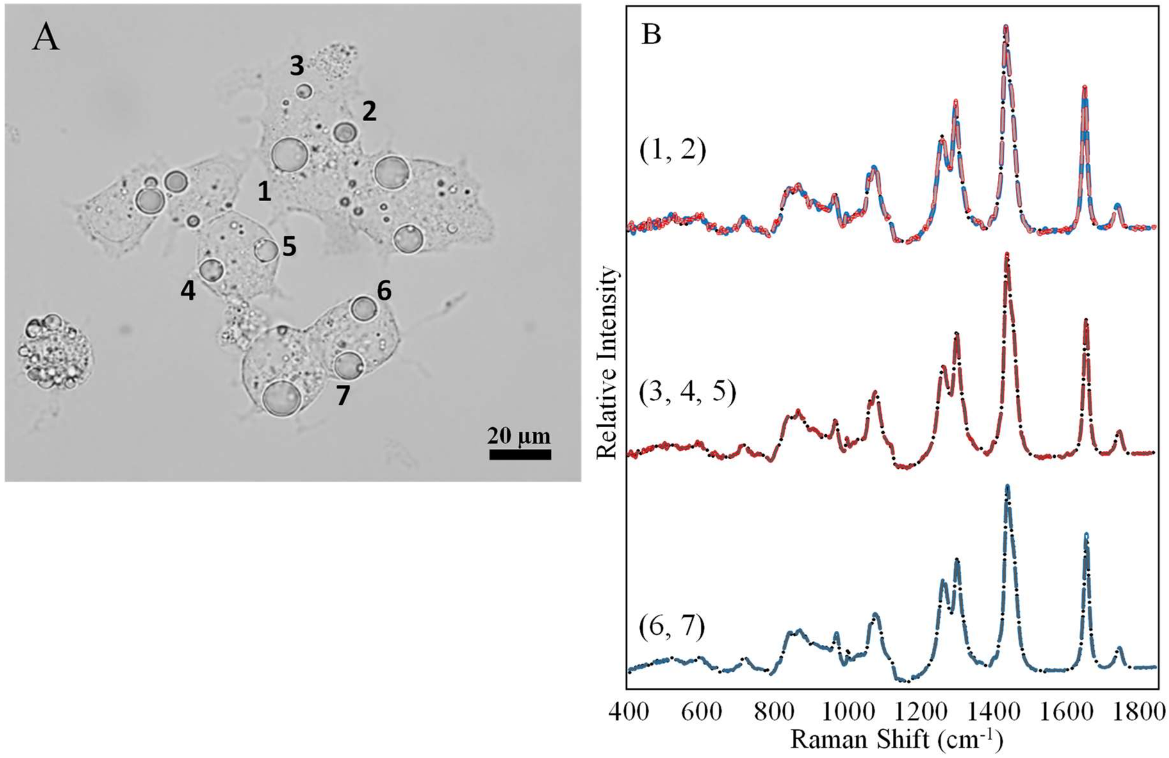

Supplementary Figure S3. Optical microscopy image of HepG2 cells cultured with linoleic acid on day 5 (A). Spectra (B) are obtained at the lipid droplets numbered 1-7. The spectra obtained in each cell are overlapped very well.
